# Photocatalytic Depolymerization of Commercial Polymethacrylates via a Solvent-Independent Pathway

**DOI:** 10.1021/jacs.6c03972

**Published:** 2026-07-10

**Authors:** Hyun Suk Wang, Victoria Lohmann, Nghia P. Truong, Athina Anastasaki

**Affiliations:** Laboratory of Sustainable Polymers, Department of Materials, 111950 ETH Zurich, Vladimir-Prelog-Weg 5, 8093 Zurich, Switzerland

## Abstract

Closed-loop chemical recycling of polymers with all-carbon backbones is an important component of plastic circularity but often requires either high temperatures (>400 °C) or noncommercial “designer” polymers. Although the feasibility of depolymerizing commercial poly­(methyl methacrylate) at ≤150 °C was recently demonstrated, a large excess of (chlorinated) solvents was deemed essential to act as a radical source. Here, we report a novel and generalizable methodology that uses diverse commercial organic and inorganic photocatalysts to decouple catalytic reactivity from the solvent. This solvent-independent approach also allows for precise control over the radical flux in nonchlorinated media, achieving near-quantitative yields (>95%) even for challenging polymers like poly­(benzyl methacrylate) and poly­(2,2,2-trifluoroethyl methacrylate) that were previously difficult to process and depolymerize. Notably, our method is compatible with phase-changing solvents (liquid during reaction, solid at room temperature), which resolves the separation–temperature trade-off and allows straightforward, quantitative collection of regenerated monomers from a solid medium.

## Graphical abstract

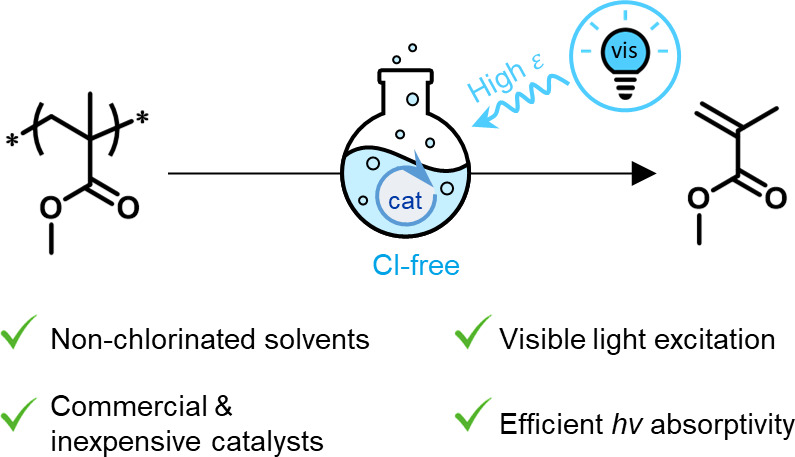

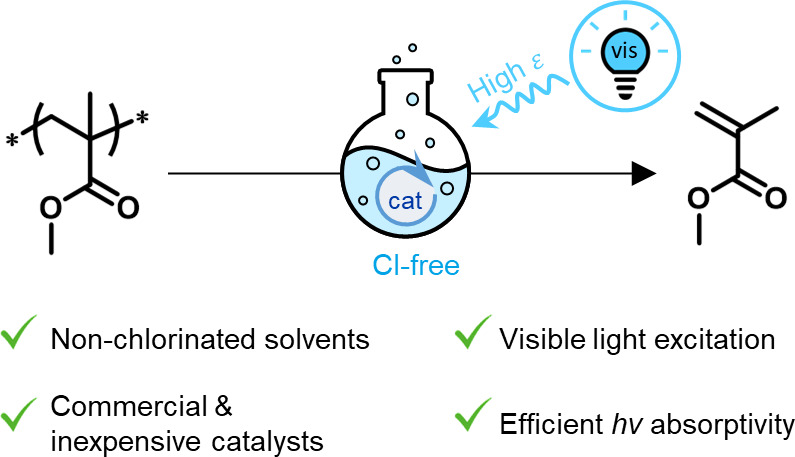

## Introduction

Plastics are an indispensable conundrum of today’s society due to their durability and chemical inertness, making recycling a pressing area of research. Mechanical recycling of these materials is the least energetically demanding and most conceptually straightforward strategy. However, mechanical recycling cannot, alone, be a permanent solution due to the accumulation of undisclosed additives, property weakening via hybridization, and chemical deterioration of the polymer chain.^1,2^ On the other hand, chemical recycling via degradation,^3–5^ repurposing/upcycling^6–10^ and depolymerization (i.e., the reversion of polymer to monomer)^1,11–13^ are promising complementary approaches, the latter enabling the reconstruction virgin-grade materials in every cycle, thereby creating an ideal closed-loop system.

The depolymerization of vinyl polymers is challenging due to their all-carbon backbones but still energetically accessible. For example, the heat of polymerization (Δ*H*_*p*_) of methyl methacrylate (MMA) is around −13 kcal/mol, resulting in a bulk ceiling temperature (*T*_c_) of ∼220 °C.^14^ However, postsynthesis, PMMA is kinetically trapped from depolymerization and requires temperature significantly above the *T*_c_ (typically >400 °C) to regenerate the polymeric radical intermediate through which depropagation proceeds.^15^ Although pyrolysis is straightforward and efficient, byproduct generation motivates the development of depolymerization approaches that reduce the kinetic barrier and thereby the reaction temperature. To reduce this kinetic barrier, polymers containing labile moieties on the chain terminus,^16–35^ pendant group,^36–40^ or backbone^3^ have been employed, either in bulk or solution, significantly reducing the depolymerization temperature. Early reports by the groups of Raus^41^ and Gramlich^42^ documented the reversibility of atom transfer radical polymerization (ATRP)^43–45^ and reversible addition–fragmentation chain-transfer (RAFT)^46–48^ polymerization of bulky methacrylates, while Ouchi and co-workers^49^ demonstrated that chlorine-capped PMMA can be depolymerized with a ruthenium catalyst. Matyjaszewski and co-workers pushed the boundaries of ATRP-type depolymerizations with various Cu- and Fe-catalyzed systems in solution and bulk.^19–23,25^ On the RAFT front, the Sumerlin group reported the solution and bulk depolymerization of polymethacrylates via thermolytic and photolytic terminal C–S cleavage.^16–18^ Other advances in RAFT-type depolymerizations include flow depolymerizations by the Junkers’ group^31,32^ and microwave-triggered depolymerizations by the Boyer group.^50^ Besides labile chain-ends, labile pendant groups have also been employed to trigger depolymerization. For example, Sumerlin demonstrated that phthalimide groups could trigger a sequential decarboxylation→depolymerization via thermolytic N–O cleavage.^36–39,51^ Backbone-attached C–Cl bonds through α-chloroacrylate comonomers have also been reported to trigger depolymerization.^100^

Despite these notable advances, these depolymerization approaches cannot be applied to current commercial materials as they rely on noncommercial “designer” polymers containing preinstalled functionalities. Furthermore, such niche materials pose severe limitations such as molecular weight dependence, reduced thermal processing stability, and nontolerance of acrylate comonomer incorporation, all of which are detrimental to real-world applications.^52^ Thus, these depolymerization approaches are not suitable to tackle the annual production of 4 million metric tons of commercial PMMA.^53^ Toward this end, Sumerlin and co-workers demonstrated an elegant method to retrofit N–O bonds into unfunctionalized PMMA via either mechanochemistry^38^ and esterification^37^ to endow depolymerizability at reduced temperatures. Although directly applicable to commercial materials, these methods required an additional postfunctionalization step to retrofit a weak link into the polymer.

Recently, we reported a one-step depolymerization approach for commercial PMMA through a photochemical backbone-initiated pathway, resulting in near-quantitative (>95%) depolymerization and concurrent chain scission (Figure 1).^52^ Unlike previous approaches which relied on a weak bond trigger (e.g., C–S, C–X, and N–O bonds), this depolymerization was initiated by hydrogen atom transfer (HAT) of the unactivated aliphatic backbone C­(sp^3^)–H (BDE_C–H_ = 96–101 kcal/mol), enabling direct unzipping of commercial PMMA at reduced temperatures. Key to this one-step depolymerization was the gradual generation of highly reactive Cl^•^ as the HAT agent (BDE_HCl_ = 103 kcal/mol) directly from the solvent. However, this approach necessitated large quantities of chlorinated solvents (e.g., 1,2-dichlorobenzene) due to their vanishingly low absorptivity in the 365–415 nm regime. This represented an overwhelming excess of chlorine atoms relative to the polymer repeat unit ([Cl]/[MMA] > 1000) when, in principle, only substoichiometric amounts should be required. Simply using substoichiometric quantities of these chlorinated species in a separate nonchlorinated solvent (i.e., a mixed solvent system) was not deemed a viable strategy due to their aforementioned low absorptivity (Figure S3). It is worth noting that a recent study by Husband et al. reaffirmed that chlorinated solvents, such as 1,2-dichlorobenzene, are required to maximize depolymerization efficiency.^54^ Further attempts to use nonchlorinated solvents, including benzonitrile, showed initial promise but led to substantially reduced monomer conversions to approximately 28–47% even under harsh, high-intensity UV irradiation and higher temperatures (175 °C). The source of hydrogen atom transfer agents in this nonchlorinated system remains unclear while the required UV photolysis of benzonitrile poses a risk of generating highly toxic hydrogen cyanide. These limitations ultimately restrict the ability of these solvent-derived radical approaches to achieve quantitative depolymerization and decouple catalytic reactivity from the solvent.

**Figure 1:**
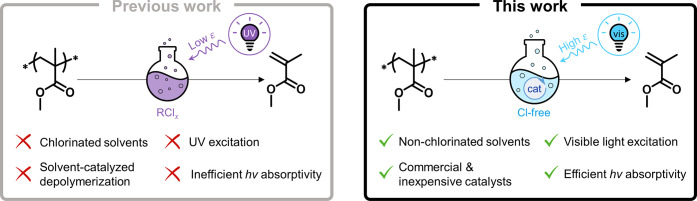
Photocatalytic depolymerization of poly­(methyl methacrylate) in nonchlorinated solvents.

Eliminating chlorinated solvents, or more broadly the need to use solvent-derived radicals, is an urgent task from a regulations standpoint but requires an overhaul of the depolymerization methodology. Specifically, three critical criteria must be met: first, a photocatalyst that can generate Cl^•^ with the same reactivity as those generated from 1,2-dichlorobenzene. Second, a compatible nonchlorinated solvent for HAT, photochemical reactions, and high temperatures (e.g., > 100 °C). Third, comparable flux of Cl^•^ to suppress biradical termination that will compete with depropagation. In this work, we aim to develop a novel catalytic depolymerization system satisfying all these criteria.

## Results and Discussion

For the catalyst, FeCl_3_/NBu_4_Cl was selected due to its ability to generate Cl^•^ via ligand-to-metal charge transfer (LMCT) and low cost.^55–57^ Benzonitrile was employed as the solvent as a high boiling point (bp 191 °C) equivalent of MeCN, the most widely employed solvent for HAT/C–H activation reactions. A benzonitrile solution of PMMA (initial repeat unit concentration [RU]_0_ = 50 mM) and 0.01 equiv of FeCl_3_/NBu_4_Cl was irradiated with blue LEDs (λ_max_ = 460 nm) at 150 °C (Figure S1). After 24 h, a dramatic decrease in the *M*_n_ (250 kDa to 12 kDa) and a depolymerization conversion of 85% by SEC (80% by ^1^H NMR, only MMA observed as product) was observed (Figures S2 and S4), strongly resembling the characteristic scission-depolymerization behavior of our previous solvent-catalyzed system.^52^ Notably, under nondeoxygenated conditions, only 3% depolymerization occurred while scission still proceeded, presumably due to rapid quenching of depropagating chains by molecular oxygen (Figure S5). A range of amine salts and halogen ligands were subsequently screened, demonstrating successful depolymerization under a range of conditions and greater dependency on the halide ligand rather than the amine salt (Figure S6). Furthermore, both commercial grade and additive-containing Plexiglas (red dye) could be depolymerized to similar conversions (80–81% by SEC, 73–76% by ^1^H NMR) with 0.01 equiv of FeCl_3_/NBu_4_Cl, demonstrating relevance to real-world products (Figures S7–S10). Additionally, solvent reuse was demonstrated by adding an extra batch of Plexiglas and 0.01 equiv of FeCl_3_/NBu_4_Cl directly into the crude reaction mixture, resulting in comparable depolymerization conversions (77%) in the second cycle (Figure S11). These promising results indicated the possibility of completely eliminating chlorinated solvents for backbone-initiated depolymerization while maintaining relatively high monomer conversion. Even at 20-fold higher polymer loadings ([RU]_0_ = 1 M), the final conversion was not significantly impacted, reaching 69% (68% by ^1^H NMR) in the presence of just 0.002 equiv of catalyst (Table S1). It is worth noting that the reported theoretical equilibrium monomer concentration at 150 °C is ∼700 mM, and thus monomer yields closely match theoretical yields.

Encouraged by these results, we proceeded to address the third criterion (i.e., optimal Cl^•^ flux) to maximize conversion. Although a wide range of catalyst loadings (0.001–0.02 equiv) were employed, comparable final conversions of 80–83% (78–80% by ^1^H NMR) were reached, with higher catalyst concentrations merely accelerating the reaction (Figure S12, Table S2). Curiously, further increasing the catalyst concentration from 0.02 to 0.1 equiv resulted in a reversal in the trend, with lower final conversions and slower kinetics observed. SEC analysis revealed that both chain scission and depolymerization were always faster at higher catalyst loadings up to 0.02 equiv, but above this loading the rate of depolymerization was retarded while chain scission continued to accelerate (Figure S13). This peculiar trend suggested that high catalyst concentrations incur premature radical quenching of the chain-end radical by chlorine and/or other carbon-centered radicals. If this were indeed the case, we posited that regulating Cl^•^ concentration may enhance the final depolymerization conversion. To test this theory, a stock solution of the catalyst was gradually fed at a rate of 0.005 equiv/h (50 μL/h) into a benzonitrile solution of PMMA being irradiated with blue LEDs at 150 °C (see Supporting Information for experimental details). In the early stages of the reaction, depolymerization proceeded slowly due to the low initial catalyst concentration but, unlike previous batch reactions, continued proceeding to >95% conversion in the same total time (Figure 2a, Figure S14a). This highlighted the importance of Cl^•^ flux in such backbone-initiated depolymerizations, akin to the increased levels of biradical termination during conventional radical polymerization at high radical concentrations. This hypothesis was further corroborated by the similar reaction kinetics to batch reactions at higher catalyst feed rates (Figure S14b) and highlights the importance of taking caution in generalizing solvent effects in HAT-triggered depolymerization systems.^54^

**Figure 2:**
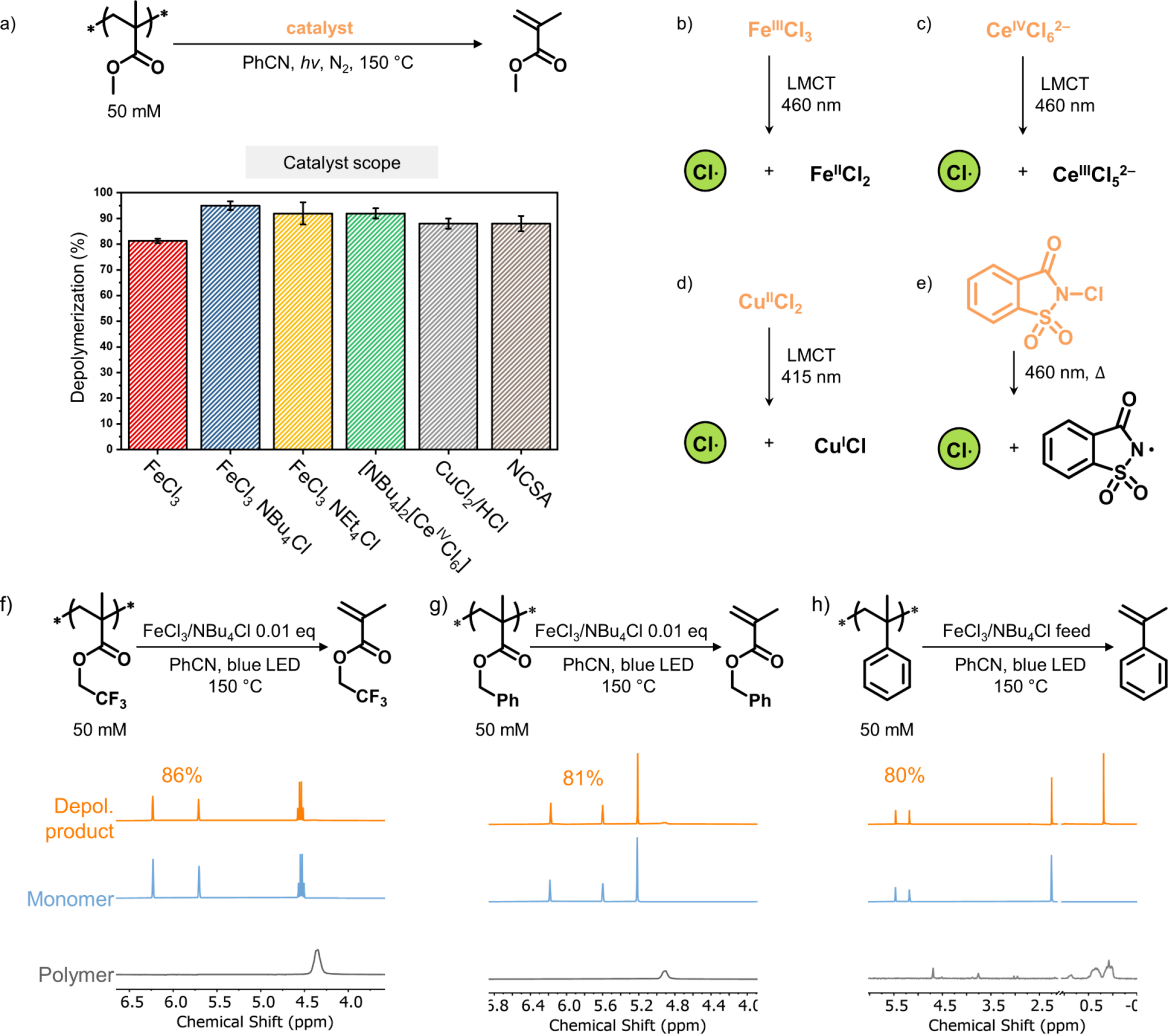
(a) Depolymerization of PMMA in PhCN at 150 °C using various catalysts as the Cl^•^ source. (b)–(e) Photochemical generation of Cl^•^ from (b) Fe^III^, (c) Ce^IV^, (d) Cu^II^, and (e) N-chlorosaccharin. (f)–(h) Depolymerization of (f) poly­(2,2,2-trifluoroethyl methacrylate), (g) poly­(benzyl methacrylate), and (h) poly­(α-methylstyrene) using Fe^III^ catalysts.

To further explore the catalyst scope beyond iron centers, [Ce^IV^Cl_6_]­[NBu_4_]_2_, Cu^II^Cl_2_/HCl, and N-chlorosaccharin (NCSA) were also tested as they are frequently used for small-molecule HAT reactions.^58–60^ With 0.001–0.04 equiv of Ce^IV^, the final conversion plateaued at 53—68% by SEC, indicating the generalizability of this photocatalytic depolymerization strategy (Table S3). Like the Fe^III^ system, while batch reactions with varying catalyst loadings did not improve the final conversion, catalyst feeding led to a significant enhancement, reaching 92% in a similar time frame (Figure 2a, Figure S15), further highlighting the importance of Cl^•^ flux. Similar trends were observed with Cu^II^, with 59—78% conversion by SEC reached with 0.002—0.1 equiv of catalyst (Table S4) and 88% by catalyst feeding (Figure 2a, Figure S16). Finally, NCSA was tested as an organic photocatalyst that can produce Cl^•^ via N–Cl cleavage. Like the trend seen in the metal-centered catalysts, batch reactions in the presence of 0.01–0.1 yielded a maximum conversion of 62% (61% by ^1^H NMR) (Table S5) whereas gradual feeding of NCSA substantially boosted the conversion to 88% by SEC (Figure 2a, Figure S17). Overall, these results demonstrate that the current photocatalytic depolymerization approach is generalizable to many inorganic and organic catalytic systems in a nonchlorinated solvent. Our findings also highlight the importance of gradual feeding as an efficient strategy to boost depolymerization conversion.

To investigate whether the backbone-initiated depolymerization could be expanded beyond PMMA and possibly other polymer classes, including those that do not contain ester functionality, we tested the FeCl_3_/NBu_4_Cl-catalyzed reaction on poly­(2,2,2-trifluoroethyl methacrylate) (PTFEMA), poly­(benzyl methacrylate) (PBzMA), and poly­(α-methylstyrene) (PAMS). Notably, PTFEMA is insoluble in dichlorobenzene but fully soluble in benzonitrile, highlighting an additional benefit of expanding the solvent scope beyond chlorinated solvents. At 0.01 equiv of catalyst, PTFEMA could be depolymerized to 86% TFEMA, determined by ^1^H NMR, demonstrating the robustness of the photocatalytic depolymerization methodology (Figure 2f, Figures S18 and S19). Next, we tested PBzMA as it contains benzylic hydrogens neighboring the ester oxygen, rendering it highly susceptible to HAT and thus competitive with the backbone C–H for the Cl^•^. Despite the presence of a weak pendant C–H, PBzMA could be depolymerized to 81% BzMA (determined by ^1^H NMR) under the same conditions, possibly because depropagation is a rapid unimolecular chain reaction that minimizes the effect of the competing benzylic C–H activation, further evidencing the robustness of the methodology (Figure 2g, Figures S18 and S19). Finally, a polymer without an ester pendant, PAMS, was tested under the same conditions to confirm a nondecarboxylative pathway. Indeed, while a lower conversion (32–49% by ^1^H NMR) was reached with 0.002–0.04 equiv of catalyst (Figures S18–S20, Table S6), feeding the catalyst during the reaction led to an improved conversion of 80% by ^1^H NMR (Figure 2h), confirming the backbone-initiated pathway.

Despite these promising results in nonchlorinated solvents, the methodology had intrinsic limitations stemming from the use of liquid solvent to energetically drive depolymerization because postdepolymerization, monomer separation from organic solvents becomes a considerable challenge. As the difference in boiling point is typically small, careful optimization of fractional distillation conditions is required to obtain pure monomer, especially at high viscosities and low monomer concentrations. We hypothesized that a phase-changing solvent could overcome this seemingly intrinsic purification-temperature trade-off by being liquid during depolymerization and solid at around room temperature, allowing straightforward solid–liquid separation while maintaining the benefit of a solution depolymerization. Important criteria for the solvent are the absence of weak C–H bonds and the ability to solubilize PMMA. To this end, we employed diphenyl ether (mp = 25–27 °C, bp = 258 °C) as a phase-changing solvent and attempted depolymerization with FeCl_3_/NBu_4_Cl as the catalyst (see Supporting Information for experimental details). At [RU]_0_ = 500 mM and 0.004 equiv of catalyst, a conversion of 57% determined by ^1^H NMR was achieved albeit at a temperature of 170 °C ([M]_eq_ ≈ 1300 mM) (Figure 3). Similar results were also observed when PBzMA was depolymerized under the same conditions (Figure S21). Although the reaction does not reach the theoretical quantitative conversion, the remarkably low catalyst content required (0.4 mol %) highlights the practicality of the photocatalytic depolymerization. As a control experiment, we conducted the depolymerization in the absence of either the catalyst or light irradiation, and observed no meaningful depolymerization and scission under these conditions (Figure S22). Upon cooling the solution to ∼20 °C in a water bath, the solution solidified, enabling straightforward vacuum distillation of the regenerated liquid MMA (>99% purity by ^1^H NMR) without the need to optimize pressure. Importantly, 95% of the regenerated MMA could be collected in one distillation step (566 mg regenerated, 538 mg collected in receiving flask) highlighting the advantages of phase-changing solvents in monomer recovery. The recovered MMA could be repolymerized into PMMA in the presence of a radical initiator, demonstrating the circularity of the methodology (Figure S23).

**Figure 3:**
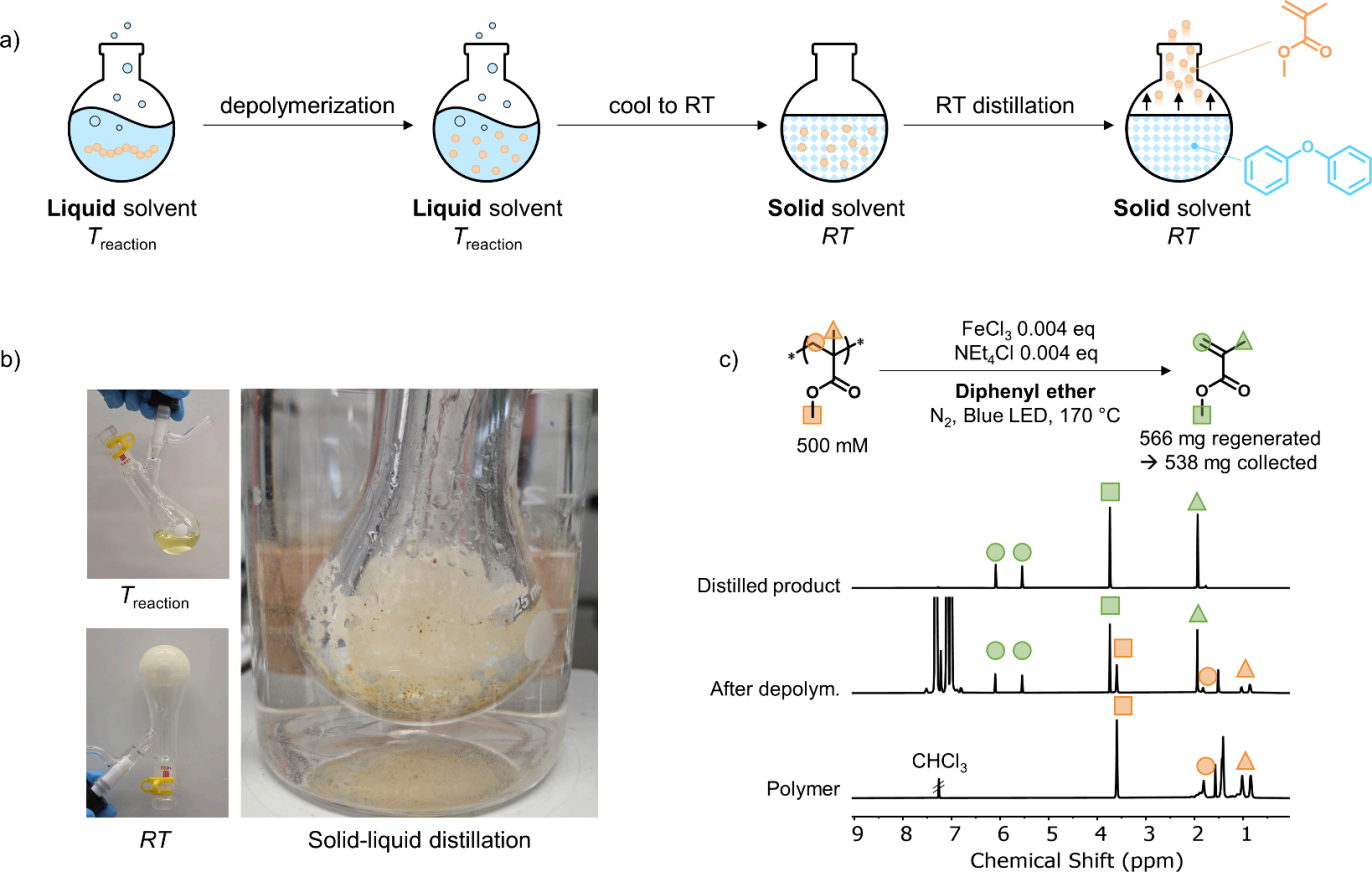
(a) Schematic of the depolymerization in a phase-changing solvent and subsequent monomer distillation from the solid medium. (b) Image of the reaction mixture at 170 °C, room temperature, and during vacuum distillation of the regenerated MMA. (c) ^1^H NMR spectra of the PMMA, crude reaction mixture after depolymerization, and the distilled product (99% purity).

## Conclusion

To summarize, we report a novel, general, and photocatalytic depolymerization approach for polymethacrylates in nonchlorinated solvents. Unlike the previous approach where an enormous excess of a chlorine source (i.e., the chlorinated solvent, [Cl]/[MMA] > 1000) was required due to the low absorptivity at 365–415 nm, the current approach requires catalytic amounts of chlorine sources (as low as [Cl]/[MMA] = 0.004) for sufficient generation of Cl^•^, enabling quantitative depolymerization and complete circumvention of chlorinated media. Notably, the photocatalytic approach is compatible with a variety of inorganic and organic catalysts, ranging from Fe^III^, Cu^II^, Ce^IV^, and N–Cl compounds, and polymer side chains, demonstrating its generalizability. Finally, expanding the solvent scope to a nonchlorinated phase-changing solvent (diphenyl ether) allows both depolymerization at subpyrolytic temperatures and straightforward separation of the regenerated monomer from a solid medium. Moving forward, it will be essential to address the scalability challenges inherent in photoreactions (e.g., light penetration and reactor setup) and, ultimately, investigating the orthogonality of the depolymerization in mixed waste streams.

## Supplementary Material



## Notes

General information, experimental procedures, ^1^H NMR spectra, SEC traces (PDF)
