# Supplementary material for: Photocatalytic Depolymerization of Commercial Polymethacrylates via a Solvent-Independent Pathway
Source: J Am Chem Soc. 2026 Jul 10;148(28):29814–20. doi: 10.1021/jacs.6c03972 (PMC13397555; doi:10.1021/jacs.6c03972)
Supplement: Supplementary file 1 [file ja6c03972_si_001.pdf]

Supporting Information

Photocatalytic Depolymerization of Commercial Polymethacrylates via a Solvent-Independent Pathway

Hyun Suk Wang[1], Victoria Lohmann[1], Nghia P. Truong[1], Athina Anastasaki[1*]

[1]Laboratory of Sustainable Polymers, Department of Materials, ETH Zurich, Vladimir-Prelog-Weg 5, Zurich, Switzerland

*Correspondence: athina.anastasaki@mat.ethz.ch

Table of Contents

Methods 1

- **Materials.** 1
- **NMR spectroscopy.** 1
- **Size-exclusion chromatography (SEC).** 1
- **Synthesis of PMMA by free radical polymerization.** 1
- **Synthesis of PBzMA and PTFEMA by free radical polymerization.** 2
- **Determination of monomer conversion during polymerization.** 2
- **Backbone-initiated depolymerization of PMMA, PTFEMA, PBzMA, and Plexiglas.** 2
- **Recycling of benzonitrile for subsequent Plexiglas depolymerization.** 2
- **Backbone-initiated depolymerization of** 3
- **Photoreactor setup.** 3
- **Determination of depolymerization conversion.** 4
- **Depolymerization via catalyst feeding.** 4
- **Backbone-initiated depolymerization of PMMA with diphenyl ether as the phase-changing solvent.** 5
- **Distillation of MMA after depolymerization in diphenyl ether.** 5

Table of Figures

Figure S1. Illustrative description of the reaction setup for main-chain-initiated depolymerization. 4
Figure S2. (a) SEC trace and (b) ^{1}H NMR spectrum ($CDCl_3$) of free radically synthesized PMMA (M_n = 250 kDa, Đ = 2.13). 6
Figure S3. Depolymerization of PMMA in mixture of 1,2-dichlorobenzene (DCB) and benzonitrile under violet LED light irradiation (415 nm) at 150 °C. Chlorine radicals are generated directly from DCB. 6
Figure S4. (a) SEC trace and (b) ^{1}H NMR spectrum after depolymerization of PMMA synthesized by free radical polymerization. $FeCl_3/NBu_4Cl$ was used as the chlorine radical source in benzonitrile. 7
Figure S5. (a) SEC trace before and after depolymerization of PMMA under non-deoxygenated conditions. $FeCl_3/NBu_4Cl$ was used as the chlorine radical source in benzonitrile. 7
Figure S6. Depolymerization conversions after 24 h in the presence of various Fe catalysts. Conversions are based on SEC analyses 8
Figure S7. (a) SEC trace and (b) ^{1}H NMR spectrum ($CDCl_3$) of commercial grade PMMA (M_n = 255 kDa, Đ = 4.08). 9
Figure S8. Depolymerization of purchased, commercial grade PMMA. (a) SEC trace before and after depolymerization and (b) ^{1}H NMR spectrum after depolymerization. 9
Figure S9. (a) SEC trace and (b) ^{1}H NMR spectrum ($CDCl_3$) of additive-containing red Plexiglas (M_n = 363 kDa, Đ = 2.87). 10
Figure S10. Depolymerization of purchased, additive-containing red Plexiglas (a) SEC trace before and after depolymerization and (b) ^{1}H NMR spectrum after depolymerization. 10
Figure S11. SEC traces of two depolymerization cycles in the same solvent. After the first cycle of depolymerization ($[RU]_0$ = 50 mM in benzonitrile, 0.01 eq $FeCl_3/NBu_4Cl$, 150 °C, blue light, 24 h), the same amount of Plexiglas and catalyst was added directly to the reaction mixture for a second cycle of depolymerization. 11
Figure S12. Depolymerization conversions after 20 h at various Fe catalyst loadings. Conversions are based on SEC analyses 11
Figure S13. M_n of remaining PMMA after 24 h of depolymerization in the presence of various equivalents of $FeCl_3/NBu_4Cl$. Reaction conditions: $[RU]_0$ = 50 mM, 150 °C, blue LED irradiation. 12
Figure S14. Effect of constant feeding of $FeCl_3/NBu_4Cl$ into a PMMA solution in PhCN ($[RU]_0$ = 50 mM, 150 °C) under blue LED irradiation. A 25 mM catalyst stock solution was used for the feeding experiment. (a) Depolymerization kinetics with and without feeding catalyst at 50 μL/h, and (b) depolymerization kinetics at various feed rates. 13
Figure S15. Backbone-initiated depolymerization of PMMA in PhCN under blue LED irradiation via feeding of $[NBu_4]_2[CeCl_6]$ (25 mM stock solution) at a rate of 50 μL/h. 14
Figure S16. Backbone-initiated depolymerization of PMMA in PhCN under blue LED irradiation via feeding of $CuCl_2$/HCl (25 mM stock solution) at a rate of 50 μL/h. 15
Figure S17. Backbone-initiated depolymerization of PMMA in PhCN under blue LED irradiation via feeding of N-chlorosaccharin (25 mM stock solution) at a rate of 50 μL/h. 16
Figure S18. Full ^{1}H NMR spectra of the depolymerization of (a) poly(2,2,2-trifluoroethyl methacrylate), (b) poly(benzyl methacrylate), and (c) poly(α-methylstyrene) using Fe^{III} catalysts. 17

Figure S19. SEC traces before and after the depolymerization of (a) poly(2,2,2-trifluoroethyl methacrylate), (b) poly(benzyl methacrylate), and (c) poly(α-methylstyrene) using Fe^{III} catalysts. 17
Figure S20. ^{1}H NMR spectra of reaction mixture after the iron-catalyzed backbone-initiated depolymerization of poly(α-methylstyrene) in PhCN under blue LED irradiation. 18
Figure S21. (a) SEC traces of PBzMA before and after depolymerization in diphenyl ether and (b) ^{1}H NMR spectrum of the crude reaction mixture after depolymerization. Reaction conditions: $[RU]_0$ = 500 mM in diphenyl ether, 0.004 eq $FeCl_3/NBu_4Cl$, blue light irradiation, 170 °C 19
Figure S22. SEC traces of PMMA before and after attempted depolymerization in the absence of either catalyst or light ($FeCl_3/NBu_4Cl$ present in the latter). Reaction conditions: $[RU]_0$ = 500 mM in diphenyl ether, 0.004 eq or no $FeCl_3/NBu_4Cl$, with or without blue light irradiation, 170 °C. 19
Figure S23. SEC traces of PMMA synthesized via free radical polymerization of the MMA that was collected via depolymerization of PMMA in diphenyl ether and subsequent distillation from the cooled, solidified solvent. Reaction conditions: MMA:AIBN = 4800:1, MMA:MeCN = 1:2 v/v, 70 °C, 15 h. M_n = 232,000 g/mol and $Đ$ = 2.25. 20

Table of Tables

Table S1. Depolymerization of PMMA under various repeat unit concentrations ($[RU]_0$) in PhCN with $FeCl_3/NBu_4Cl$ as the catalyst under blue LED irradiation at 150 °C. 8
Table S2. Depolymerization conversions after 20 h at various Fe catalyst loadings. 12
Table S3. Backbone-initiated depolymerization using Ce^{IV} as the chlorine radical source. 14
Table S4. Backbone-initiated depolymerization using Cu^{II} as the chlorine radical source. 15
Table S5. Backbone-initiated depolymerization using N-chlorosaccharin as the chlorine radical source. 16
Table S6. Iron-catalyzed backbone-initiated depolymerization of poly(α-methylstyrene) in PhCN under blue LED irradiation. 18

Methods

Materials. All materials were purchased from either Sigma Aldrich, Fisher Scientific, or Tokyo Chemical Industries unless otherwise stated. Monomers were filtered through basic alumina before use. Poly(α-methylstyrene) was purchased from Agilent. $[NBu_4]_2[Ce(IV)Cl_6]$ was synthesized using a previously reported procedure.[1] Red Plexiglas was purchased from Roehm AG.

NMR spectroscopy. ^{1}H-NMR spectra were recorded on either a Bruker Avance-300 spectrometer or Bruker Avance III HD 500 MHz NMR spectrometer equipped with a 5 mm liquid-state Prodigy™ CryoProbe spectrometer using acetone-d_6, dimethyl sulfoxide-d_6, or $CDCl_3$ as the NMR solvent. Chemical shifts are given in ppm downfield from tetramethylsilane and referenced to residual solvent proton signals.

Size-exclusion chromatography (SEC). SEC was measured on a Shimadzu equipment comprising a CBM-20A system controller, LC-20AD pump, SIL-20A automatic injector, 10.0 μm bead-size guard column (50 x 7.5 mm) followed by three KF-805L columns (300 x 8 mm, bead size: 10 μm, pore size maximum: 5000 Å), SPD-20A ultraviolet detector, and an RID-20A differential refractive index detector. The column temperature was maintained at 40 ℃ using a CTO-20A oven. The flow rate was set to 1 mL/min with *N*, *N*-dimethylacetamide (DMAc, Acros, HPLC grade, with 0.03 w/v LiBr) as the eluent. Molecular weights were determined relative to poly(methyl methacrylate) standards with molecular weights ranging from 5,000 to 1.5×10^6 g/mol (Agilent Technologies). All SEC samples were dissolved in DMAc and passed through 0.45 μm filters prior to analysis.

Synthesis of PMMA by free radical polymerization. Into a 250 mL round bottom flask, 24 mL of MMA (22.6 g, 225 mmol, 4000 equiv) was added to 45 mL acetonitrile. 9.25 mg of AIBN (56.3 μmol, 1 equiv) was transferred to the flask. A stirrer bar was added to the solution and the flask was sealed with a septum and subsequently deoxygenated by nitrogen bubbling for 15 min. Polymerization was conducted in an oil bath at 70 °C for 15 h with a 400-rpm stirring rate. Samples were taken periodically under a nitrogen blanket for ^{1}H-NMR analysis and passed through a syringe filter (0.45 μm PTFE membrane) prior to SEC analysis. The polymerization was stopped by removing the reaction from the oil bath and removing the septum. The polymer was purified by precipitating in methanol. Polymer samples were dried under reduced pressure at least overnight prior to use.

Synthesis of PBzMA and PTFEMA by free radical polymerization. A representative example for PBzMA is described here. Into a 25 mL round bottom flask, 5.0 mL of benzyl methacrylate (5.2 g, 29.5 mmol, 4000 equiv) was added to 5 mL toluene. 1.2 mg of AIBN (7.3 μmol, 1 equiv) was transferred to the flask. A stirrer bar was added to the solution and the flask was sealed with a septum and subsequently deoxygenated by nitrogen bubbling for 15 min. Polymerization was conducted in an oil bath at 70 °C for 15 h with a 400-rpm stirring rate. Samples were taken periodically under a nitrogen blanket for ^{1}H-NMR analysis and passed through a syringe filter (0.45 μm PTFE membrane) prior to SEC analysis. The polymerization was stopped by removing the reaction from the oil bath and removing the septum. The polymer was purified by precipitating in methanol and then fractionally precipitating in a dichloromethane/methanol mixture. PTFEMA was precipitated in n-hexane. Polymer samples were dried under reduced pressure at least overnight prior to use. (PBzMA: M_n = 449,000 g/mol, $Đ$ = 1.71, PTFEMA: 353,700 g/mol, $Đ$ = 1.94)

Determination of monomer conversion during polymerization. Monomer conversions were determined by NMR spectroscopy. The monomer vinyl signals were compared to the combined polymer and monomer ester signals (-CH_3 for PMMA, -CH_2- for rest of the polymers used).

Backbone-initiated depolymerization of PMMA, PTFEMA, PBzMA, and Plexiglas. PMMA depolymerizations conducted at $[RU]_0$ = 50 mM and 0.001 eq $FeCl_3$/NBu_4Cl will be used as a representative example. The only difference between the polymethacrylates is the amount of polymer added to the reaction. In a 20 mL glass tube with a magnetic stir bar, 25 mg of PMMA was dissolved in 5 mL PhCN. To this solution, 25 μL of a catalyst stock solution (100 mM $FeCl_3$/NBu_4Cl in MeCN) was added. An aliquot of 200 μL was sampled from the reaction to prepare the initial SEC sample (PhCN removed via gentle air flow and subsequently dissolved in 1.1 mL DMAc for SEC analysis). The reaction tube was subsequently sealed with a rubber septum and deoxygenated by nitrogen bubbling for 15 min. Subsequently, the tube was submerged into a 150 ˚C oil bath until the top level of the solution was submerged ~1 cm below the surface of the oil bath. The reaction solution was left in the oil bath for at least 3 min to allow temperature equilibration before being initiated by irradiating the solution with blue LED strips surrounding the oil bath. The setup of the photoreactor is described below (Figure S1). Reactions were left for 24 h before removal from the photoreactor. Some reactions were performed in a glass Schlenk tube with a Teflon plug valve to obtain ^{1}H NMR samples to analyze monomer regeneration.

Recycling of benzonitrile for subsequent Plexiglas depolymerization. In a 20 mL glass tube with a magnetic stir bar, 25 mg of Plexiglas was dissolved in 5 mL PhCN. To this solution, 25 μL of a catalyst stock solution (100 mM $FeCl_3$/NBu_4Cl in MeCN) was added. The reaction tube was

subsequently sealed with a rubber septum and deoxygenated by nitrogen bubbling for 15 min. Subsequently, the tube was submerged into a 150 ˚C oil bath until the top level of the solution was submerged ~1 cm below the surface of the oil bath. The reaction solution was left in the oil bath for at least 3 min to allow temperature equilibration before being initiated by irradiating the solution with blue LED strips surrounding the oil bath. After 24 h, the reactor was removed from the oil bath and left to cool to room temperature. Subsequently, the septum was removed and 25 mg of Plexiglas and 25 uL of the same catalyst stock solution was added directly to the reaction mixture and the reaction tube was sealed with a rubber septum. After the Plexiglas had fully dissolved, the solution was deoxygenated by bubbling with nitrogen for 15 min. Subsequently, the tube was submerged into a 150 ˚C oil bath until the top level of the solution was submerged ~1 cm below the surface of the oil bath. The reaction solution was left in the oil bath for at least 3 min to allow temperature equilibration before being initiated by irradiating the solution with blue LED strips surrounding the oil bath. The reaction was removed after 24 h.

Backbone-initiated depolymerization of poly(α-methylstyrene). Depolymerization protocol is identical to that of PMMA at $[RU]_0$ = 50 mM, only differing in the mass of polymer added (29.6 mg PAMS in 5 mL PhCN).

Photoreactor setup. An oil bath in a transparent 400 mL glass beaker was surrounded 360° by either 6 rows of blue (460 nm) or violet (415 nm) LED strips (**Figure S1**). The distance between each LED and the reaction tube was 4.5 $\pm$ 0.5 cm. LED strips were purchased from Waveform Lighting. 415 nm LED strips are part of the realUV™ lineup whereas the blue LED strips (460 nm) are part of the SimpleColor™ lineup.

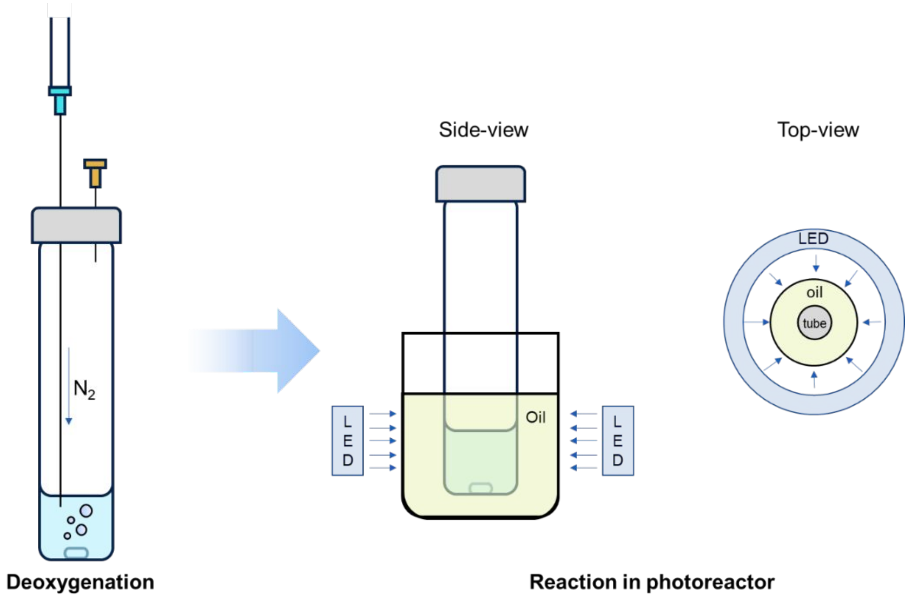

Figure S1. Illustrative description of the reaction setup for main-chain-initiated depolymerization.

Determination of depolymerization conversion. Depolymerization conversions were determined by comparing the area under the polymer peak in SEC. As the same volume of the reaction was sampled for every SEC sample, the conversion was simply calculated using the formula conversion = $(1 - (Area_t/Area_{t0}))*100$ where *t*0 and *t* denotes samples taken at time *t* = 0 h and *t*. ^{1}H NMR of the reaction mixture was used to cross-check the depolymerization conversion.

Depolymerization via catalyst feeding. In a 20 mL glass tube with a magnetic stir bar, 25 mg of PMMA was dissolved in 5 mL PhCN. The reaction tube was subsequently sealed with a rubber septum and deoxygenated by nitrogen bubbling for 15 min. Separately, 4 mL of a 25 mM $FeCl_3/NBu_4Cl$ stock solution in PhCN was prepared in a 5 mL glass vial, sealed with a rubber septum, and deoxygenated by nitrogen bubbling for 10 min. Subsequently, 1 mL of the catalyst solution was collected using a degassed needle attached to a 1 mL syringe, then wrapped in aluminium foil to prevent light penetration into the catalyst solution. The needle was then inserted through the glass tube septum and into the polymer solution under positive nitrogen pressure. The syringe was mounted onto a syringe pump (New Era Pump Systems, Inc., NE-300). The glass tube was then lowered into a 150 °C oil bath and left for at least 3 min to allow temperature equilibration. The solution was then irradiated with blue LED strips surrounding the oil bath (Figure S1) and subsequently the syringe pump was activated to start catalyst feeding. Reactions were

conducted under positive nitrogen pressure to offset pressure buildup from the feeding. Reactions were left for around 24 h before removal from the photoreactor.

Backbone-initiated depolymerization of PMMA with diphenyl ether as the phase-changing solvent. To a 25 mL Schlenk flask with a Teflon plug valve on the branch, a magnetic stir bar was added and 1 g of PMMA was dissolved in 20 mL diphenyl ether. To this solution, 400 µL of a catalyst stock solution (100 mM $FeCl_3$/NBu_4Cl in MeCN) was added and, while open, heated to 150 °C under a flow of nitrogen to evaporate the MeCN from the catalyst stock solution. Subsequently, a glass stopper was used to seal the top of the Schlenk flask the solution was degassed by three freeze-pump-thaw cycles with a final nitrogen back-fill. Subsequently, the flask was submerged into a 170 ˚C oil bath until the top level of the solution was submerged ~1 cm below the surface of the oil bath. The reaction solution was left in the oil bath for at least 5 min to allow temperature equilibration before being initiated by irradiating the solution with blue LED strips surrounding the oil bath. After 24 h, 56.6% depolymerization was achieved according to ^{1}H NMR, corresponding to 566 mg of MMA regenerated.

Distillation of MMA after depolymerization in diphenyl ether. After depolymerization, the reaction solution was cooled in a cold water bath to accelerate freezing of diphenyl ether (m.p. 25 °C). The glass stopper on the Schlenk flask was replaced with distillation bridge attached to a receiving flask. The solution was then degassed by two freeze-pump-thaw cycles and MMA distilled at room temperature into the receiving flask that was cooled by liquid nitrogen. 538 mg of MMA was collected, corresponding to 95% recovery of the regenerated MMA (566 mg regenerated according to ^{1}H NMR, 538 mg collected).

Additional Data

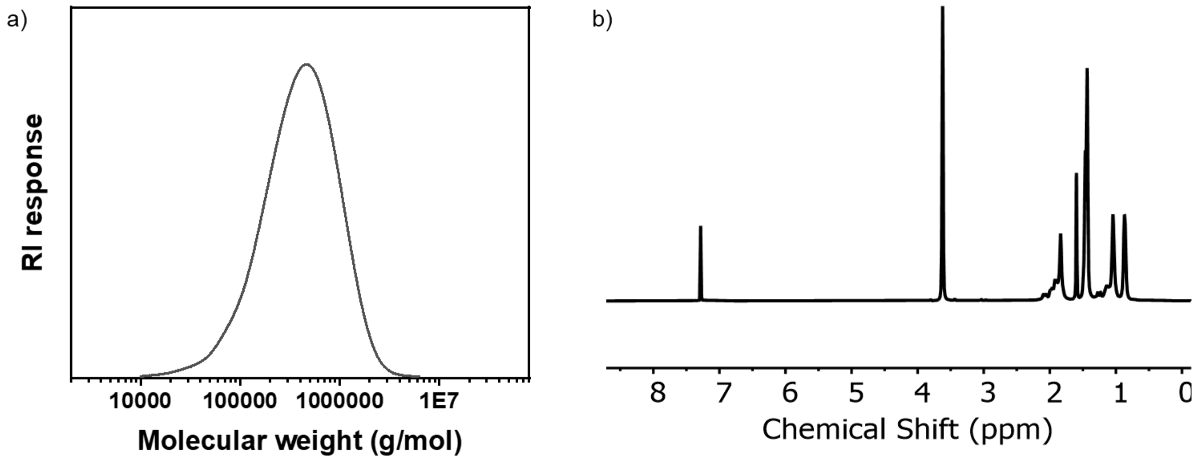

Figure S2. (a) SEC trace and (b) ^{1}H NMR spectrum ($CDCl_3$) of free radically synthesized PMMA (M_n = 250 kDa, $Đ$ = 2.13).

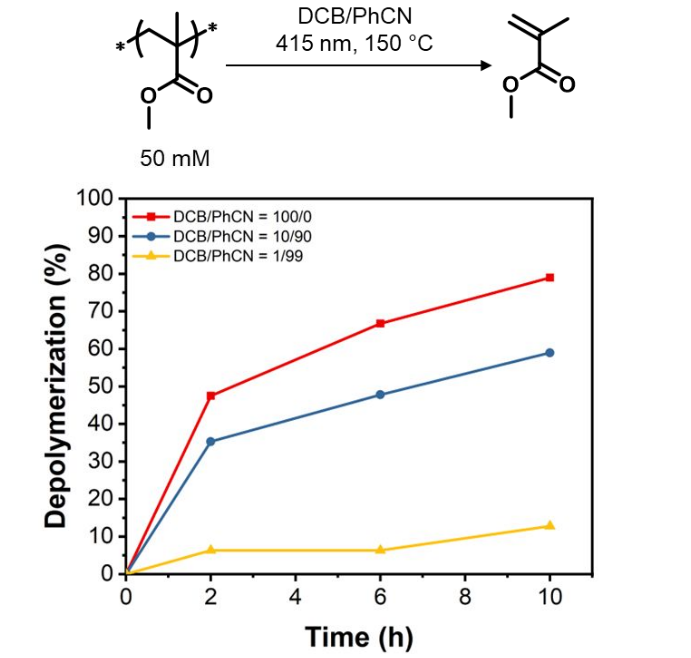

Figure S3. Depolymerization of PMMA in mixture of 1,2-dichlorobenzene (DCB) and benzonitrile under violet LED light irradiation (415 nm) at 150 °C. Chlorine radicals are generated directly from DCB.

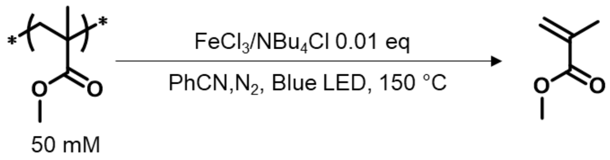

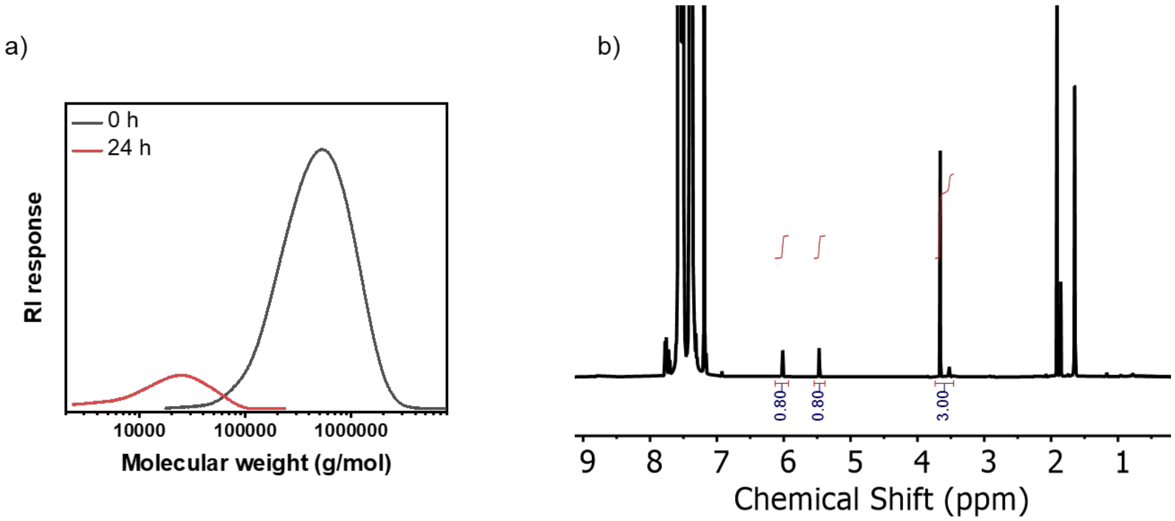

Figure S4. (a) SEC trace and (b) ^{1}H NMR spectrum after depolymerization of PMMA synthesized by free radical polymerization. $FeCl_3/NBu_4Cl$ was used as the chlorine radical source in benzonitrile.

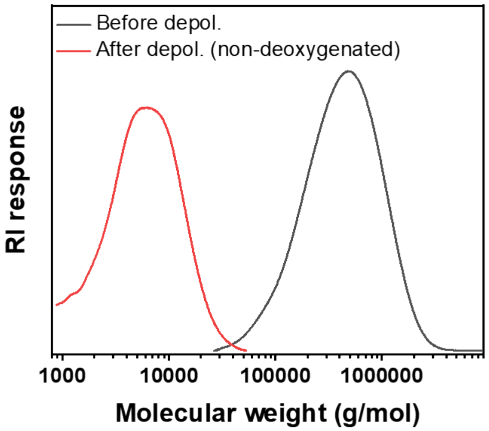

Figure S5. (a) SEC trace before and after depolymerization of PMMA under non-deoxygenated conditions. $FeCl_3/NBu_4Cl$ was used as the chlorine radical source in benzonitrile.

Table S1. Depolymerization of PMMA under various repeat unit concentrations ($[RU]_0$) in PhCN with $FeCl_3/NBu_4Cl$ as the catalyst under blue LED irradiation at 150 °C.

$[RU]_0$ (mM)	$[cat]_0$ (mM)	equiv. cat	% Depolym.^{SEC}	% Depolym.^{NMR}
100	0.5	0.005	79	75
100	2	0.02	80	76
500	0.5	0.001	67	67
500	2	0.004	78	76
1000	0.5	0.0005	59	59
1000	2	0.002	69	68

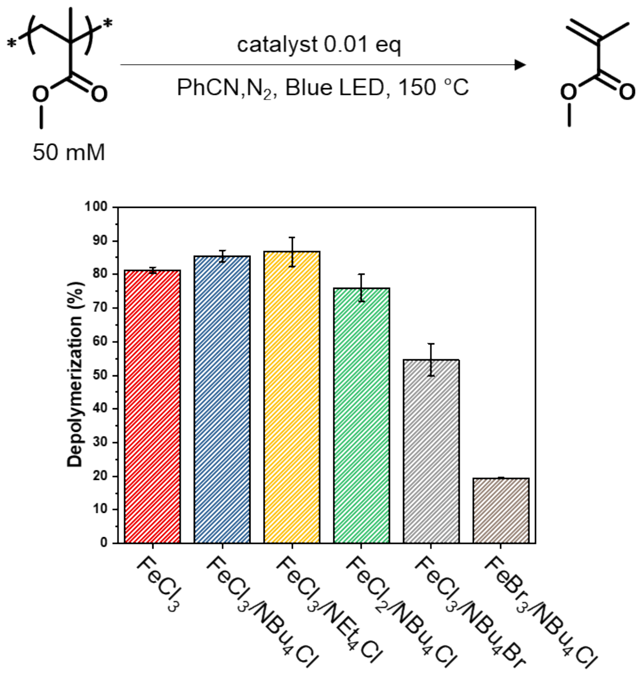

Figure S6. Depolymerization conversions after 24 h in the presence of various Fe catalysts. Conversions are based on SEC analyses.

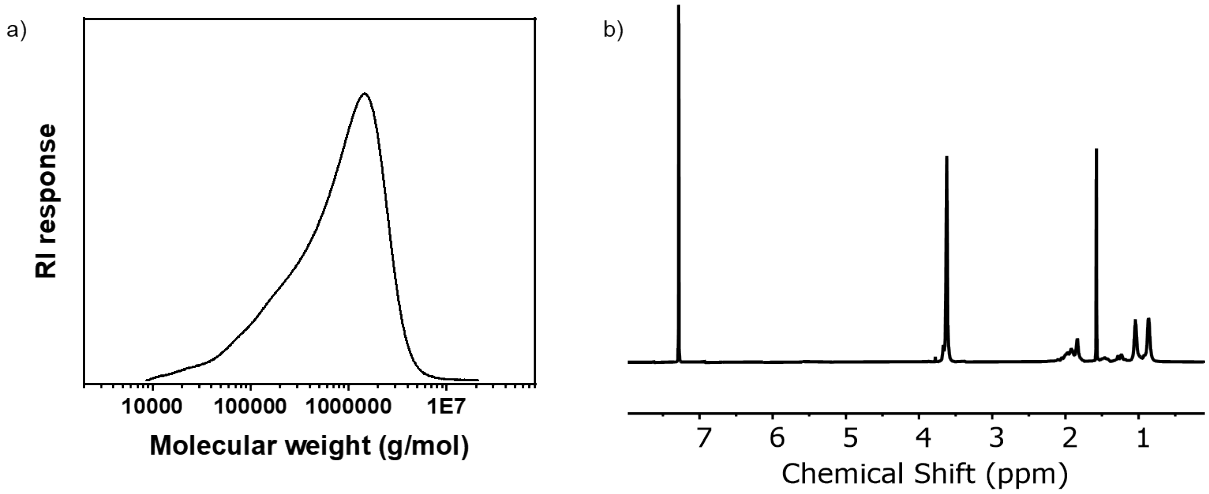

Figure S7. (a) SEC trace and (b) ^{1}H NMR spectrum ($CDCl_3$) of commercial grade PMMA (M_n = 255 kDa, $Đ$ = 4.08).

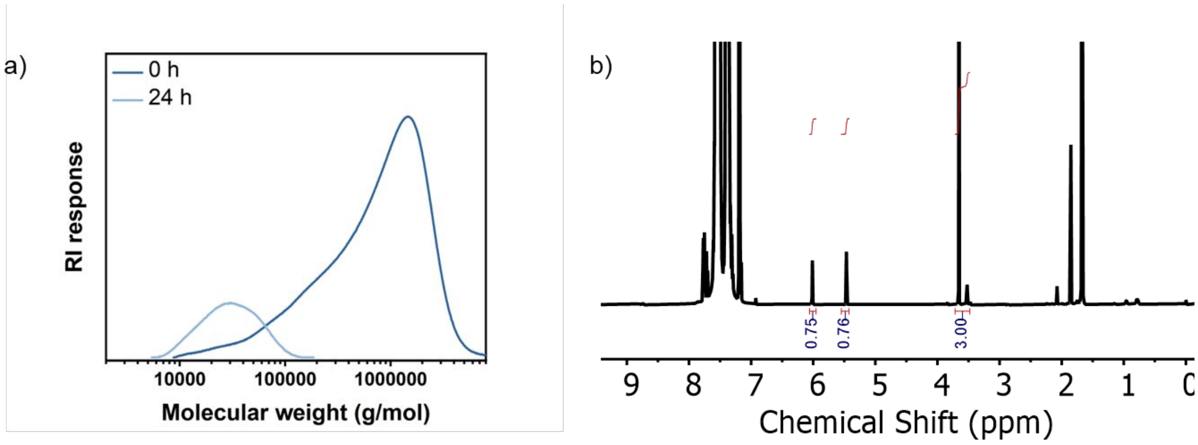

Figure S8. Depolymerization of purchased, commercial grade PMMA. (a) SEC trace before and after depolymerization and (b) ^{1}H NMR spectrum after depolymerization.

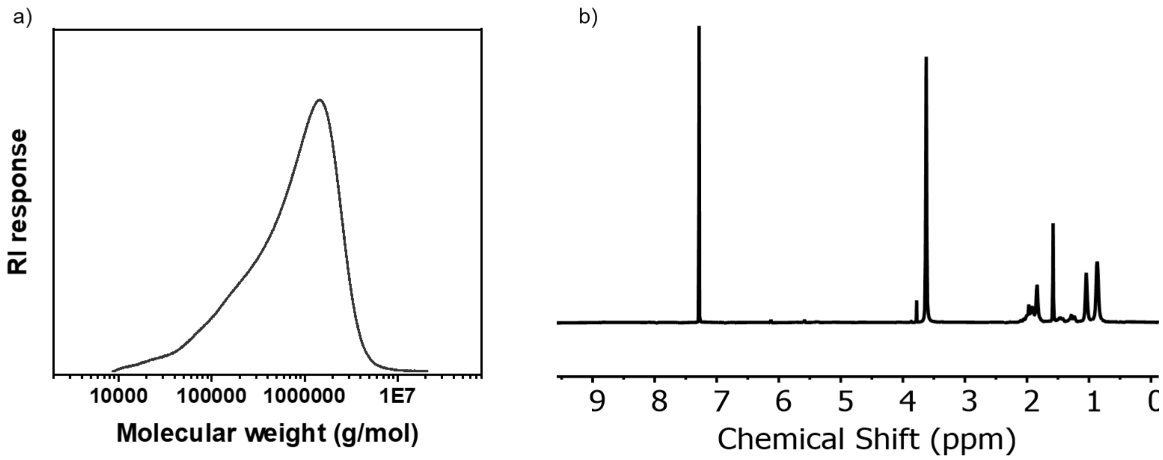

Figure S9. (a) SEC trace and (b) ^{1}H NMR spectrum ($CDCl_3$) of additive-containing red Plexiglas (M_n = 363 kDa, $Đ$ = 2.87).

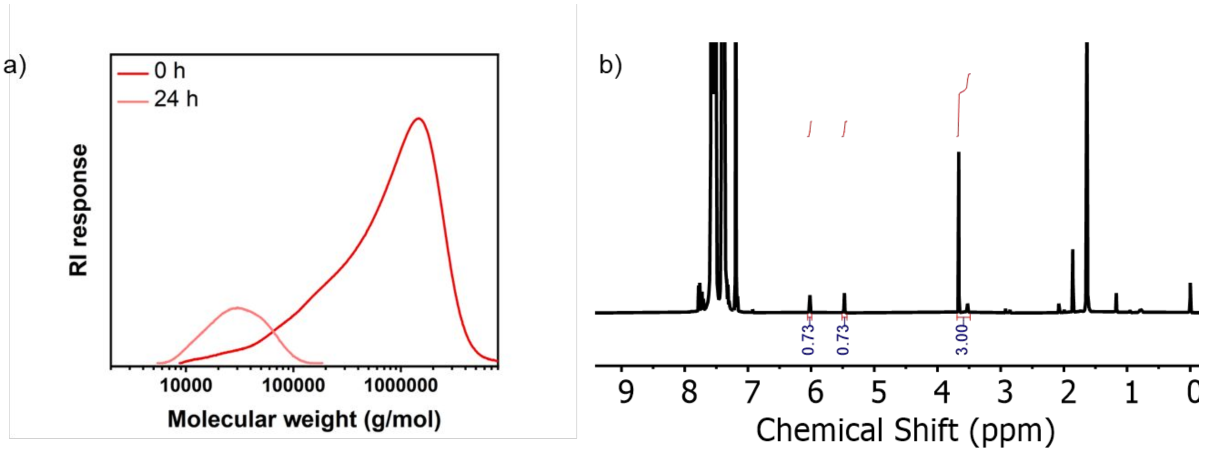

Figure S10. Depolymerization of purchased, additive-containing red Plexiglas (a) SEC trace before and after depolymerization and (b) ^{1}H NMR spectrum after depolymerization.

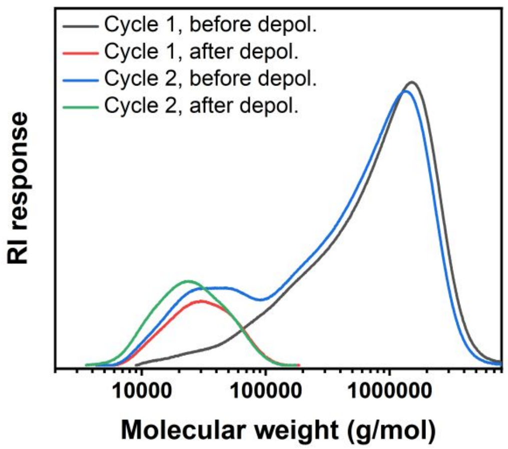

Figure S11. SEC traces of two depolymerization cycles in the same solvent. After the first cycle of depolymerization ($[RU]_0$ = 50 mM in benzonitrile, 0.01 eq $FeCl_3/NBu_4Cl$, 150 °C, blue light, 24 h), the same amount of Plexiglas and catalyst was added directly to the reaction mixture for a second cycle of depolymerization.

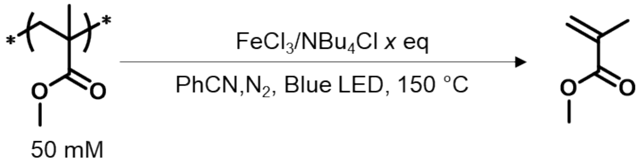

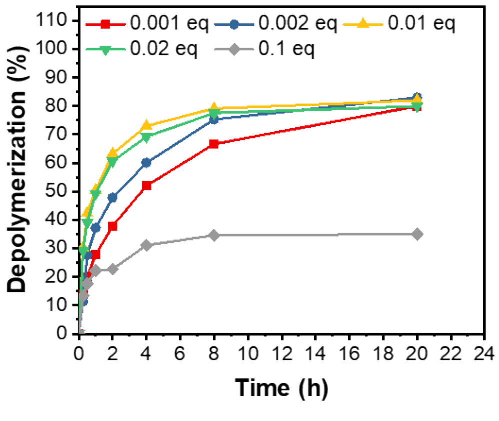

Figure S12. Depolymerization conversions after 20 h at various Fe catalyst loadings. Conversions are based on SEC analyses.

Table S2. Depolymerization conversions after 20 h at various Fe catalyst loadings.

$[RU]_0$ (mM)	$[cat]_0$ (mM)	equiv. cat	% Depolym.^{SEC}	% Depolym.^{NMR}
50	0.05	0.001	80	78
50	0.1	0.002	83	81
50	0.5	0.01	82	82
50	1	0.02	80	79
50	5	0.1	35	25

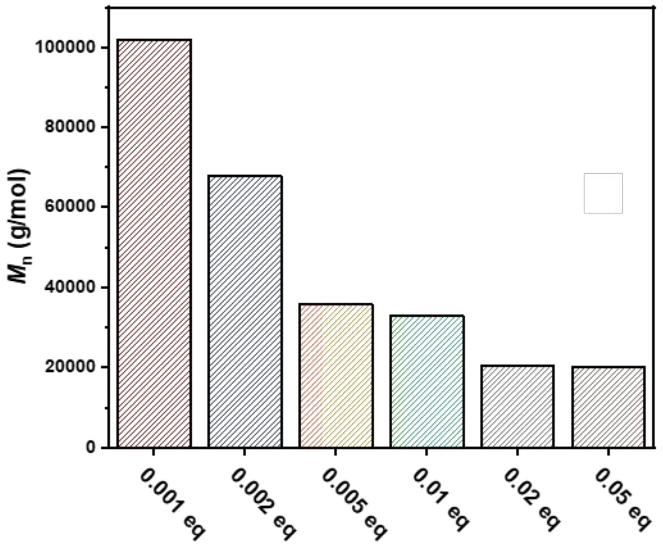

Figure S13. M_n of remaining PMMA after 24 h of depolymerization in the presence of various equivalents of $FeCl_3/NBu_4Cl$. Reaction conditions: $[RU]_0$ = 50 mM, 150 °C, blue LED irradiation.

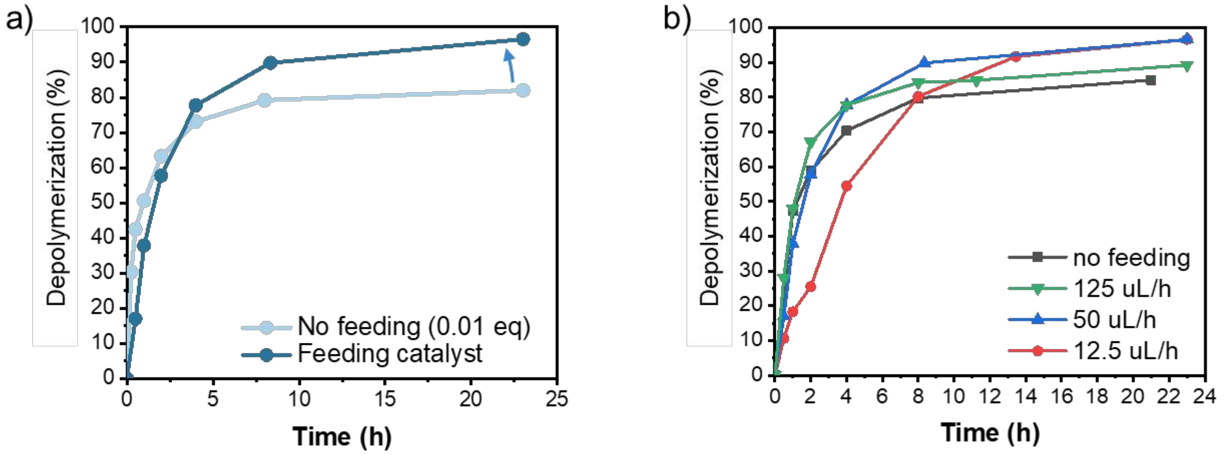

Figure S14. Effect of constant feeding of $FeCl_3$/NBu_4Cl into a PMMA solution in PhCN ($[RU]_0$ = 50 mM, 150 °C) under blue LED irradiation. A 25 mM catalyst stock solution was used for the feeding experiment. (a) Depolymerization kinetics with and without feeding catalyst at 50 μL/h, and (b) depolymerization kinetics at various feed rates.

Table S3. Backbone-initiated depolymerization using Ce^{IV} as the chlorine radical source.

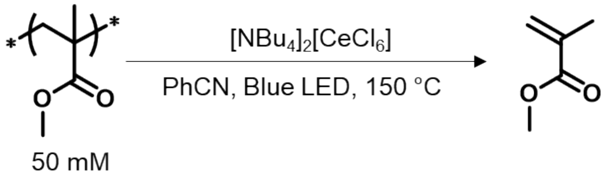

Entry	equiv. cat.	Time (h)	Depol.^{SEC}
1	$[NBu_4]_2[CeCl_6]$ (0.001)	17	53
2	$[NBu_4]_2[CeCl_6]$ (0.002)	17	62
3	$[NBu_4]_2[CeCl_6]$ (0.01)	17	68
4	$[NBu_4]_2[CeCl_6]$ (0.02)	17	65
5	$[NBu_4]_2[CeCl_6]$ (0.04)	17	66

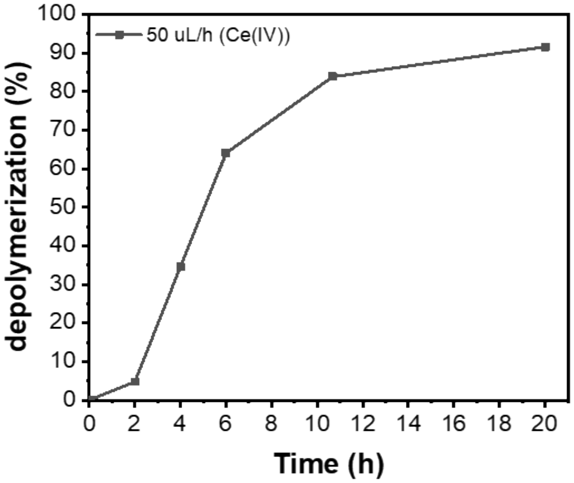

Figure S15. Backbone-initiated depolymerization of PMMA in PhCN under blue LED irradiation via feeding of $[NBu_4]_2[CeCl_6]$ (25 mM stock solution) at a rate of 50 μL/h.

Table S4. Backbone-initiated depolymerization using Cu^{II} as the chlorine radical source.

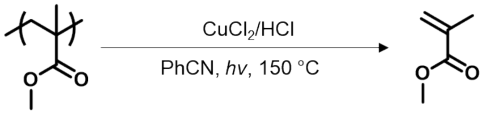

Entry	equiv. cat.	Wavelength (λ_{max})	Time (h)	Depol.^{SEC}
1	$CuCl_2/NBu_4Cl$ (0.002)	460	17	47
2	$CuCl_2/NBu_4Cl$ (0.01)	460	17	46
3	$CuCl_2/NBu_4Cl$ (0.02)	460	17	30
4	$CuCl_2$/HCl (0.002)	460	17	42
5	$CuCl_2$/HCl (0.01)	460	17	35
6	$CuCl_2$/HCl (0.02)	460	17	18
7	$CuCl_2$/HCl (0)	415	17	7
8	$CuCl_2$/HCl (0.002)	415	17	64
9	$CuCl_2$/HCl (0.01)	415	17	78
10	$CuCl_2$/HCl (0.02)	415	17	73
11	$CuCl_2$/HCl (0.1)	415	17	59

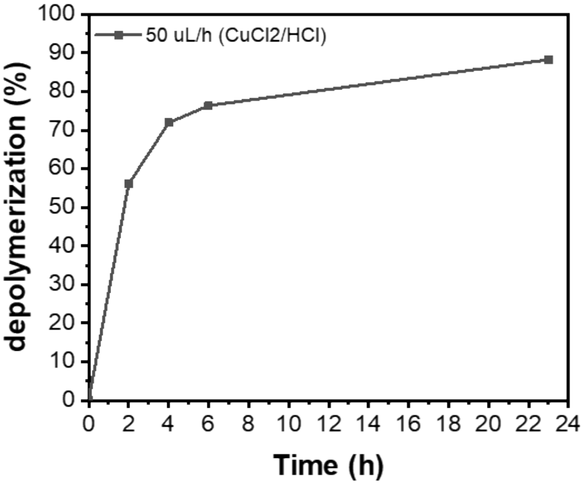

Figure S16. Backbone-initiated depolymerization of PMMA in PhCN under blue LED irradiation via feeding of $CuCl_2$/HCl (25 mM stock solution) at a rate of 50 μL/h.

Table S5. Backbone-initiated depolymerization using N-chlorosaccharin as the chlorine radical source.

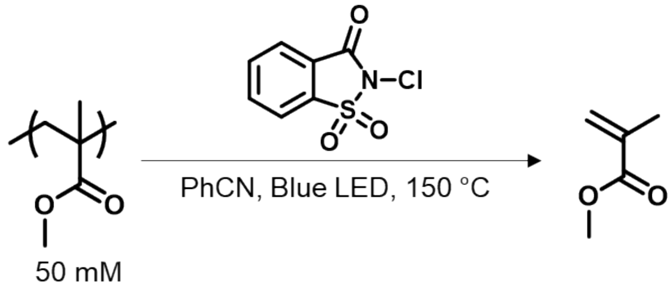

Entry	equiv. cat.	Time (h)	Depol.^{SEC}
1	N-chlorosaccharin (0.01)	16	51
2	N-chlorosaccharin (0.02)	16	62
3	N-chlorosaccharin (0.1)	16	29

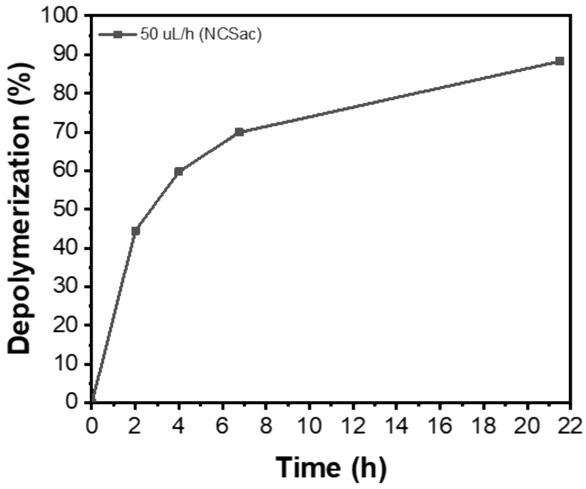

Figure S17. Backbone-initiated depolymerization of PMMA in PhCN under blue LED irradiation via feeding of N-chlorosaccharin (25 mM stock solution) at a rate of 50 μL/h.

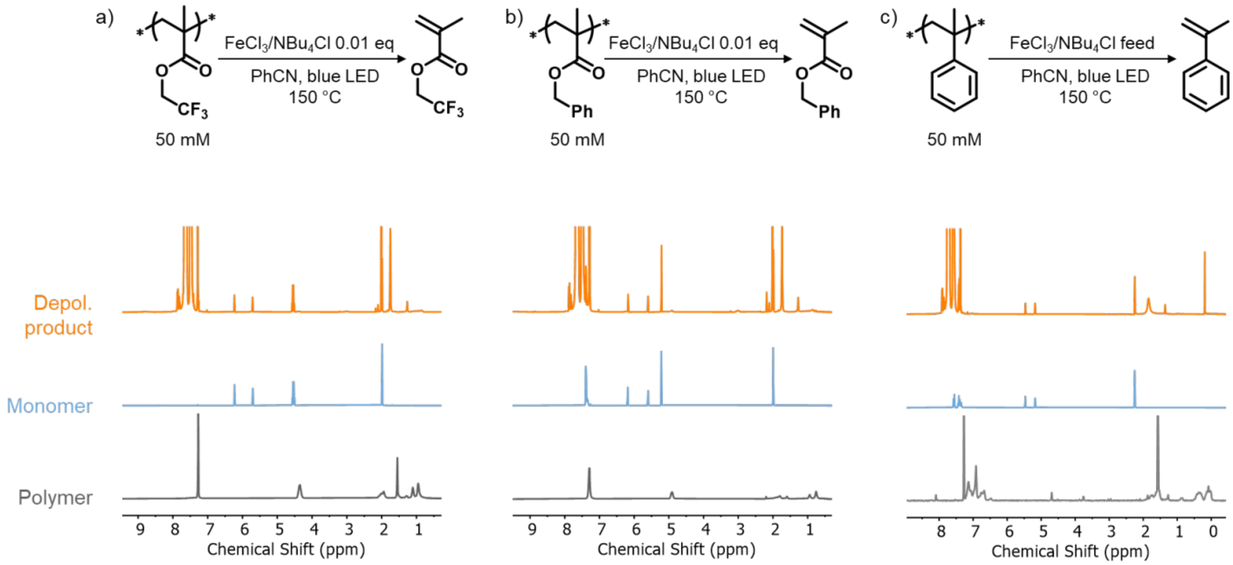

Figure S18. Full ^{1}H NMR spectra of the depolymerization of (a) poly(2,2,2-trifluoroethyl methacrylate), (b) poly(benzyl methacrylate), and (c) poly(α-methylstyrene) using Fe^{III} catalysts.

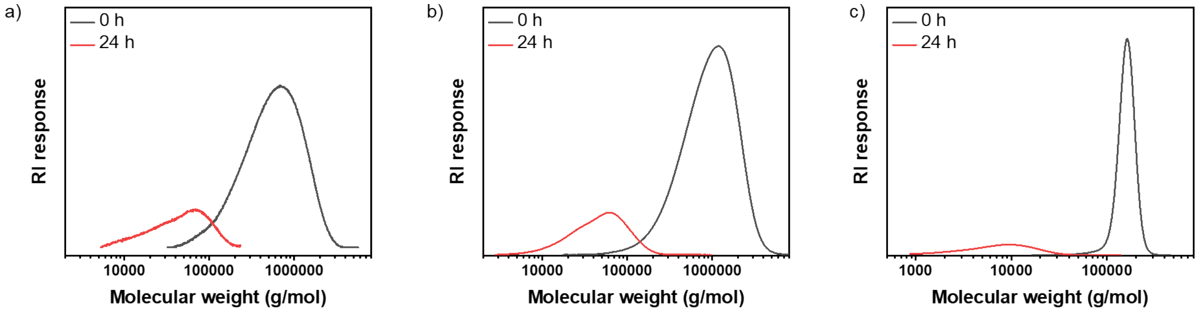

Figure S19. SEC traces before and after the depolymerization of (a) poly(2,2,2-trifluoroethyl methacrylate), (b) poly(benzyl methacrylate), and (c) poly(α-methylstyrene) using Fe^{III} catalysts.

Table S6. Iron-catalyzed backbone-initiated depolymerization of poly(α-methylstyrene) in PhCN under blue LED irradiation.

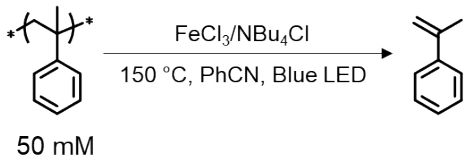

Entry	equiv. cat.	Time (h)	% Depol.^{NMR}
1	0.002	17	49
2	0.01	17	44
3	0.04	17	32
4	Feed 50 μL/h	14	80

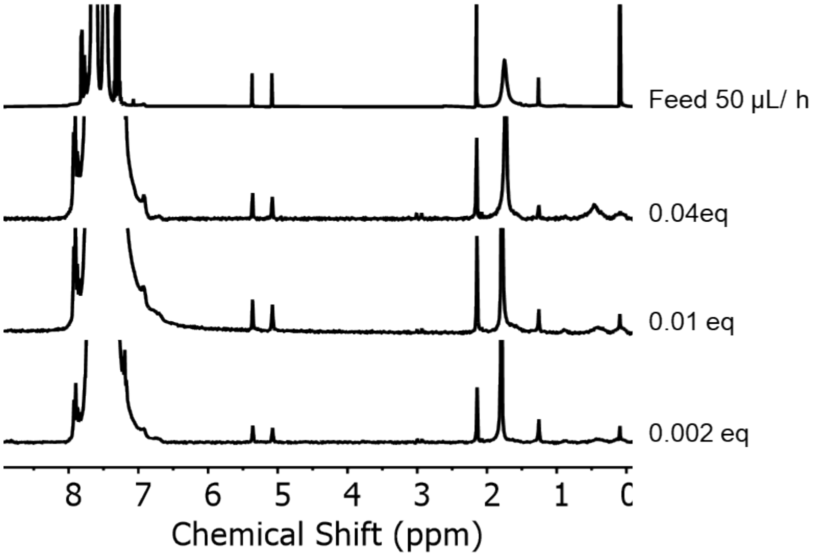

Figure S20. ^{1}H NMR spectra of reaction mixture after the iron-catalyzed backbone-initiated depolymerization of poly(α-methylstyrene) in PhCN under blue LED irradiation.

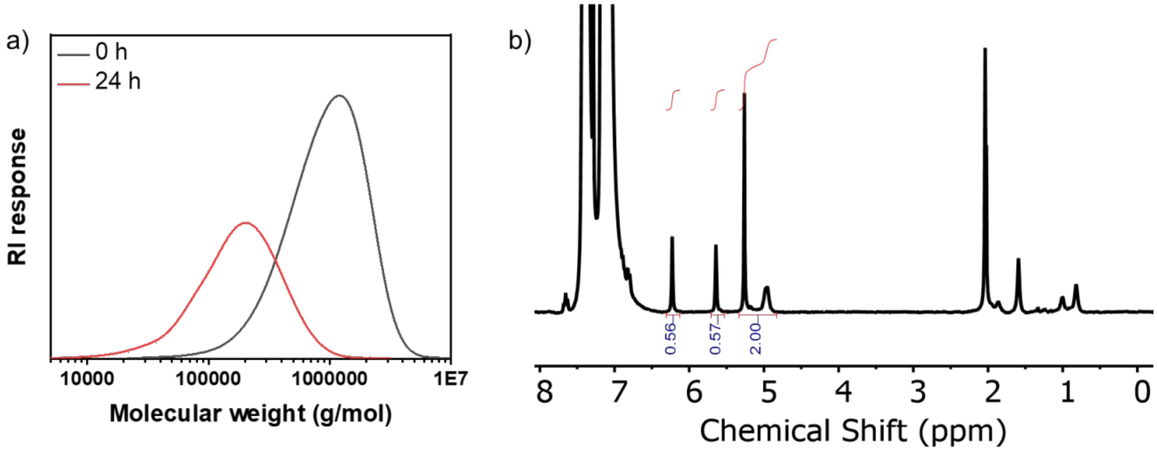

Figure S21. (a) SEC traces of PBzMA before and after depolymerization in diphenyl ether and (b) ^{1}H NMR spectrum of the crude reaction mixture after depolymerization. Reaction conditions: $[RU]_0$ = 500 mM in diphenyl ether, 0.004 eq $FeCl_3/NBu_4Cl$, blue light irradiation, 170 °C

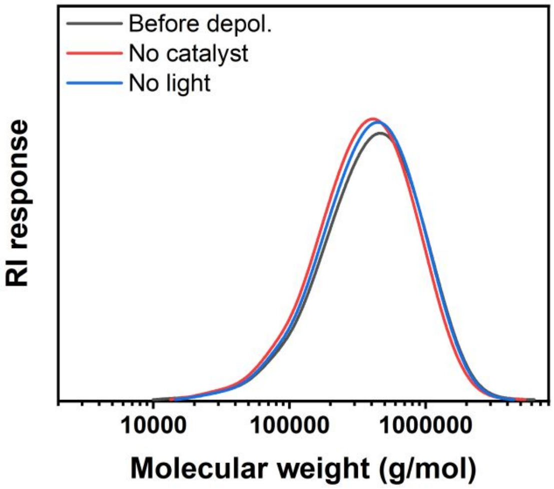

Figure S22. SEC traces of PMMA before and after attempted depolymerization in the absence of either catalyst or light ($FeCl_3/NBu_4Cl$ present in the latter). Reaction conditions: $[RU]_0$ = 500 mM in diphenyl ether, 0.004 eq or no $FeCl_3/NBu_4Cl$, with or without blue light irradiation, 170 °C.

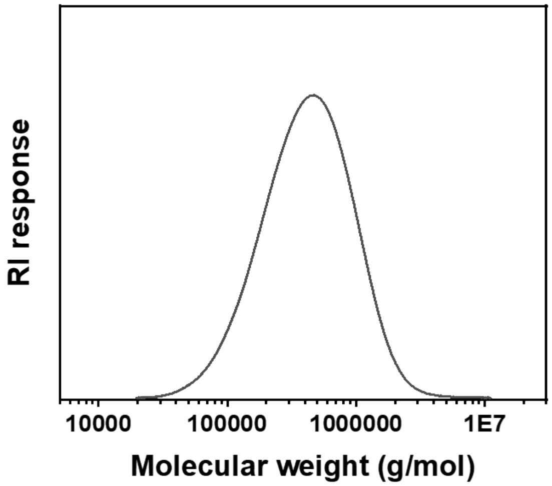

Figure S23. SEC traces of PMMA synthesized via free radical polymerization of the MMA that was collected via depolymerization of PMMA in diphenyl ether and subsequent distillation from the cooled, solidified solvent. Reaction conditions: MMA:AIBN = 4800:1, MMA:MeCN = 1:2 v/v, 70 °C, 15 h. M_n = 232,000 g/mol and $Đ$ = 2.25.

.

Reference

1. Löble, M. W.; Keith, J. M.; Altman, A. B.; Stieber, S. C. E.; Batista, E. R.; Boland, K. S.; Conradson, S. D.; Clark, D. L.; Lezama Pacheco, J.; Kozimor, S. A.; Martin, R. L.; Minasian, S. G.; Olson, A. C.; Scott, B. L.; Shuh, D. K.; Tyliszczak, T.; Wilkerson, M. P.; Zehnder, R. A., Covalency in Lanthanides. An X-ray Absorption Spectroscopy and Density Functional Theory Study of LnCl6x– (x = 3, 2). *J. Am. Chem. Soc.* **2015,** *137* (7), 2506-2523.
